# A rapid approach to investigate spatiotemporal distribution of phytohormones in rice

**DOI:** 10.1186/s13007-016-0147-1

**Published:** 2016-11-17

**Authors:** Wen-Jing Cai, Tian-Tian Ye, Qing Wang, Bao-Dong Cai, Yu-Qi Feng

**Affiliations:** Key Laboratory of Analytical Chemistry for Biology and Medicine (Ministry of Education), Department of Chemistry, Wuhan University, Wuhan, 430072 People’s Republic of China

**Keywords:** UPLC–MS/MS, Phytohormones, Spatiotemporal distribution, Rice

## Abstract

**Background:**

Phytohormones play crucial roles in almost all stages of plant growth and development. Accurate and simultaneous determination of multiple phytohormones enabled us to better understand the physiological functions and the regulatory networks of phytohormones. However, simultaneous determination of multiple phytohormones in plant is still a challenge due to their low concentrations, structural and chemical diversity, and complex matrix of plant tissues. Therefore, development of a simple and selective method for the simultaneous determination of multiple phytohormones is highly needed.

**Results:**

We developed a clean-up strategy for profiling of multiple phytohormones, which can overcome the challenge of structural and chemical diversity. By using a one-step dispersive solid-phase extraction (DSPE) combined with UPLC–MS/MS, 54 phytohormones including auxins, ABA, SA, JA, GAs and CKs were simultaneously analyzed from a single rice sample extract. Using the developed method, we investigated the spatiotemporal distribution of phytohormones in rice. The profiling of various tissues of rice at different growth stages revealed the complexity of metabolic regulation and allocations of phytohormone species.

**Conclusion:**

A rapid one-step method was developed for the simultaneous analysis of six groups of phytohormones, including cytokinins, auxins, salicylic acid, jasmonates, abscisic acid and gibberellins in a single run, using UPLC–ESI–MS/MS. The proposed method was successfully applied to investigate spatiotemporal distribution of multiple phytohormones in rice. The spatiotemporal information obtained may be helpful for better understanding of phytohormones functions throughout life cycle of rice when integrated into transcriptome and other omics data.

**Electronic supplementary material:**

The online version of this article (doi:10.1186/s13007-016-0147-1) contains supplementary material, which is available to authorized users.

## Background

Phytohormones are *a group of naturally occurring, organic substances which influence physiological processes at low concentrations* [1]. They play crucial roles in almost all stages of plant growth and development, from embryogenesis to senescence. In addition, they also regulate response of plant to biotic and abiotic stress [2]. Phytohormones have been categorized into several groups based on their structures and physiological functions, including auxins, cytokinins (CKs), abscisic acid (ABA), jasmonates (JAs), salicylates, gibberellins (GAs), ethylene (ET), brassinosteroids (BRs), polyamines, signal peptides and the more-recently-discovered hormones, strigolactones (SLs) [1]. Each class of phytohormone has characteristic biological functions. However, increasing evidence shows that multiple phytohormones can mediate plant growth and development by additive, synergistic or antagonistic actions [3–7]. Phytohormone concentration and distribution are determinants of phytohormone action [8]. Therefore, studies on phytohormone functions and regulation networks primarily rely on sensitive and high-throughput methods for quantification of endogenous phytohormones in plants. Accurate and simultaneous determination of multiple phytohormones enabled us to better understand the physiological functions and the regulatory networks of phytohormones. Hirano et al. [9] presented the dynamic changes of each phytohormone during rice microspore/pollen (MS/POL) development by analysis of endogenous levels of ABA, CKs, GAs and IAA combined with the transcriptome results in mature anther. According to spatial and temporal distribution of CKs and the related gene function assays, Rijavec et al. [10] found that CKs may perform highly contrasting roles in the filial endosperm and maternal tissues of developing seed in maize. Based on phytohormone profiling and RNA-seq analyses, Chao et al. [11] discovered the specific combination of phytohormones involved in bud differentiation and shoot growth at different time points. Hence, simultaneous profiling of multiple classes of hormones, especially integrated with the results of related gene expression profilings, is a powerful tool to reveal the mechanisms and interactions of phytohormones in different growth and development stages of plants [10].

There are two ways to get information about the concentrations of multiple phytohormones in plant samples. One is to divide the sample to multiple portions for independent analysis of multiple classes of phytohormones respectively [9]. However, this requires a large amount of plant sample, which cannot meet the increasing demand for analysis of limited amounts of plant samples, such as a tiny organ of a rice. The other way is to develop methods for simultaneous determination of multiple phytohormones in one sample. Simultaneous analysis of multiple phytohormones is challenging due to their structural and chemical diversity, and the low contents in plant samples, usually at the nanomolar level, as well as the complex plant matrix. Therefore, it’s of great significance to design a feasible strategy for simultaneous analysis of multiple phytohormones. Great efforts have been made. Additional file 1: Table S1 presents a summary of representative analytical methods for simultaneous determination of multiple phytohormones. Multiple steps involving liquid–liquid extractions or solid-phase extractions, as well as combinations of them have been used for the removal of the sample matrix and enrichment of multiple phytohormones [12–30]. Kojima et al. [12] developed a multi-step strategy for determination of 43 phytohormones including auxins, ABA, GAs and CKs. The phytohormones in rice were stepwise separated into several fractions by multiple solid-phase extraction (SPE). “MS-probe” bromocholine was used for derivatization of fractions containing auxin, ABA and gibberellins to increase the MS detection sensitivity. Subsequently, phytohormones in each fraction were, respectively, analyzed using UPLC–MS/MS [12]. Cao et al. [14] reported a method using liquid chromatography-triple quadrupole mass spectrometry (LC–MS/MS) for the profiling and quantification of 43 phytohormones and their major metabolites, including auxins, abscisic acid, jasmonic acid, salicylic acid, cytokinins and gibberellins in a single sample extract purified by binary extraction using commercial polymer anion exchange resin (PAX) and polymer cation exchange resin (PCX), respectively. Liu et al. described a method for simultaneous analysis of 24 acidic and alkaline phytohormones, in which a binary SPE using Oasis MCX cartridges for cations and Oasis MAX cartridges for anions was employed for purification and enrichment of phytohormones. Alkaline and acidic phytohormones were eluted from different SPE cartridges, respectively. The two fractions of elution were combined for UPLC–MS/MS analysis [15]. Obviously, these multiple SPE strategies were tedious and time-consuming. One-step methods can be more efficient. Recently, Meulebroek et al. [26] developed a generic extraction protocol combining an UPLC-Orbitrap-MS detection method for analysis of eight phytohormones (GA3, ABA, IAA, JA, SA, Z, BA and BL) in both tomato fruit and leaf tissue. Crude plant extract was just passed through a 30 kDa Amicon^®^ Ultra centrifugal filter unit prior to LC–MS analysis. Pan et al. described a protocol for quantitative analysis of major phytohormones in crude plant extracts by high-performance liquid chromatography–mass spectrometry. Dichloromethane was used to extract and purify seven major classes phytohormones from plant extract before LC–MS analysis [28, 31]. Although these one-step sample preparation protocols are simple and fast, the matrix effect does exist and the efficiency of purification should be further improved. Cai et al. [19] developed a method to comprehensively profile phytohormones, including 8 cytokinins (CKs), indole-3-acetic acid (IAA), abscisicacid (ABA), jasmonic acid (JA) and 10 gibberellins (GAs) by Fe_3_O_4_@TiO_2_-based magnetic solid-phase extraction coupled with ultra-performance liquid chromatography-electrospray tandem mass spectrometry (Fe_3_O_4_@TiO_2_-based MSPE-UPLC–MS/MS). Whereas, to date, these materials are not readily available in most laboratories.

In the current work, we have developed a clean-up strategy for profiling of multiple phytohormones, which can overcome the challenge of structural and chemical diversity. By using a one-step dispersive solid-phase extraction (DSPE) combined with UPLC–MS/MS, 54 phytohormones including auxins, ABA, SA, JA, GAs and CKs were simultaneously analyzed from a single rice sample extract. Using the developed method, we have investigated the spatiotemporal distribution of phytohormones in rice.

## Results and discussion

### Method for rapid quantification of phytohormones

**Fig. 1:**
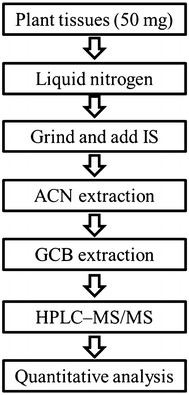
Schematic representation of the extraction and purification protocol for rapid quantification of phytohormones. *IS* internal standards

Graphitized carbon black (GCB) has been widely used in QuEChERS method for sample cleanup because it can remove chlorophyll through π–π interaction with porphyrin ring of chlorophyll [32–34]. In this study, we choose GCB for cleanup of plant samples. The overall procedure for extraction and purification of phytohormones is summarized in Fig. 1. Acetonitrile is used to extract phytohormones from plant [29], and graphitized carbon black (GCB) sorbent is employed for dispersive solid-phase extraction (DSPE) cleanup. In order to obtain an optimal extraction efficiency, three parameters including the amount of GCB, water content of sampling solution and extraction time were optimized (Additional file 1: Fig. S1). As the amount of GCB increased, more amount of analytes were adsorbed and less remained in the supernatant, resulting in decreased recoveries. When sampling in ACN, analytes would be adsorbed by GCB via hydrophilic interaction, so addition of H_2_O could improve the recoveries. Finally, 10 mg of GCB for DSPE, 80% ACN (v/v) for sampling and 3 min for extraction were employed for further experiments. Under the optimized conditions, the absolute recoveries of 54 phytohormones spiked in 80% ACN (v/v) and plant sample matrix were investigated respectively by using the proposed DSPE. The recoveries in standards were calculated by comparing standards that were extracted with standards without extraction. The recoveries in matrix samples were calculated by comparing samples that were spiked and then extracted with those, which were extracted and then spiked. Internal standards were added to the samples before injection to UPLC–MS/MS to calibrate errors of instrument detection. And the results are listed in Additional file 1: Table S3. Recoveries of most phytohormones in sample matrix were higher than in standards. This might be because that the sample matrix may block GCB binding sites so that less phytohormones were absorbed to the GCB and more phytohormones remained in the supernatant, resulting in higher recoveries in sample matrix.

**Fig. 2:**
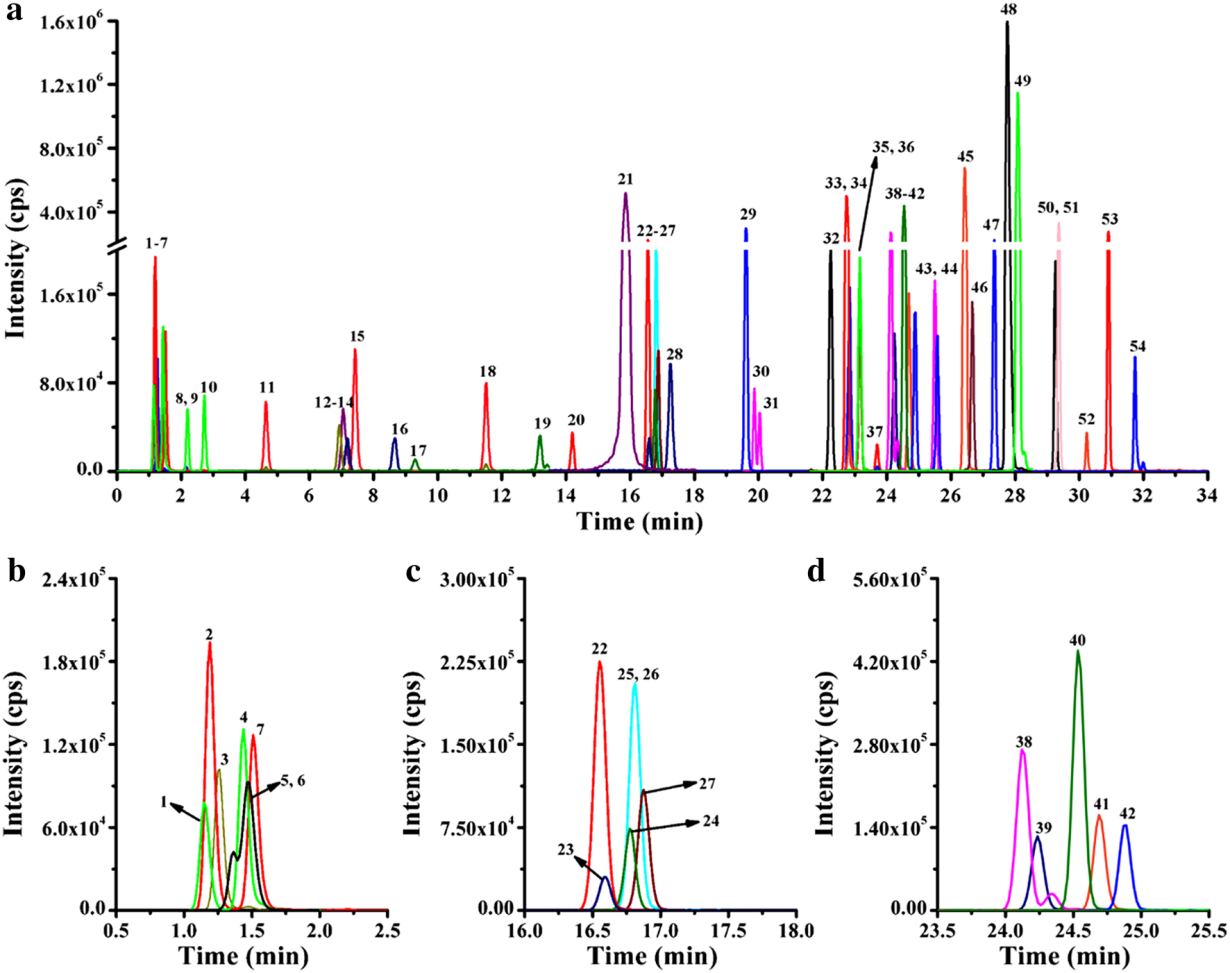
The MRM chromatograms of 54 phytohormones analyzed by UPLC–ESI–MS/MS. **a** Peak 1–54; **b** peak 1–7; **c** peak 22–27; **d** peak 38–42; 1, tZ7G; 2, tZ; 3, DZ; 4, cZOG; 5, DZ7G; 6, DZOG; 7, cZ; 8, DZ9G; 9, tZ9G; 10, cZ9G; 11, iP7G; 12, iP; 13, DZR; 14, tZR; 15, GA_8_; 16, cZR; 17, GA_29_; 18, iP9G; 19, 12OHJA; 20, GA_23_; 21, SA; 22, 2CltZ; 23, GA_3_; 24, iPR; 25, 2MeStZ; 26, 2MeScZ; 27, GA_1_; 28, IAA; 29, GA_6_; 30, 2MeStZR; 31, 2MeStZR; 32, ABA; 33, GA_13_; 34, GA_5_; 35, GA_19_; 36, GA_20_; 37, GA_44_; 38, JA; 39, IBA; 40, GA_34_; 41, 2MeSiP; 42, GA_51_; 43, GA_53_; 44, 2MeSiPR; 45, GA_7_; 46, GA_4_; 47, GA_24_; 48, JA-leu; 49, JA-phe; 50, GA_15_; 51, GA_9_; 52, 2BSiP; 53, GA_12_; 54, OPDA. The full names and abbreviations of the phytohormones can be found in “Chemicals and reagents” section

Ultra Performance Liquid Chromatography (UPLC) was employed for separation of 54 phytohormones. The chromatograms are shown in Fig. 2. Fifty-four tested analytes achieved baseline separation by UPLC, except for DZ7G and DZOG, MeStZ and MeScZ. Appropriate precursor to product ion transitions for each compound (54 molecular species) and their respective deuterium-labeled internal standards were determined by UPLC–ESI–MS/MS (Additional file 1: Table S2). Cytokinins and auxins were detected in the positive ion mode, ABA, JA, SA and gibberellins were identified in the negative ion mode. A polarity-switching mode enables the analysis of compounds with different preferred ionization modes. In order to enhance the sensitivity of UPLC–ESI–MS/MS, six separate functions were implemented in the MRM mode so that only ions eluted during the specified retention windows were monitored. The reproducibility and accuracy of the proposed method were evaluated with intra-day and inter-day measurements. The intra-day precisions were obtained with extractions of five samples over a day, and the inter-day precisions were obtained by extracting samples in continuous three days. The RSDs of inter- and intra-day precision were below 11.8%, and the relative recoveries were in the range of 80.3–120.4%, indicating good reproducibility and accuracy of the method (Additional file 1: Table S5). The limits of quantifications (LOQs) were calculated as the signal-to-noise ratios of 10:1 on standards with 3 replicate injections, ranging from 0.05 fmol for 2MeStZ to 29.92 fmol for cZOG in cytokinins, from 0.18 fmol for GA_1_ to 27.5 fmol for GA_9_ in gibberellins, 12.88 fmol for IAA, 93.29 fmol for SA and 1.12 fmol for JA (Additional file 1: Table S4). The LOQs are comparable with the majority of the methods in Additional file 1: Table S1. However, the presented method has the advantage of being faster than most other methods and can analyze multiple phytohormones in a single UPLC–ESI–MS/MS run (Additional file 1: Table S1).

### Spatiotemporal distribution of phytohormones in rice

**Fig. 3:**
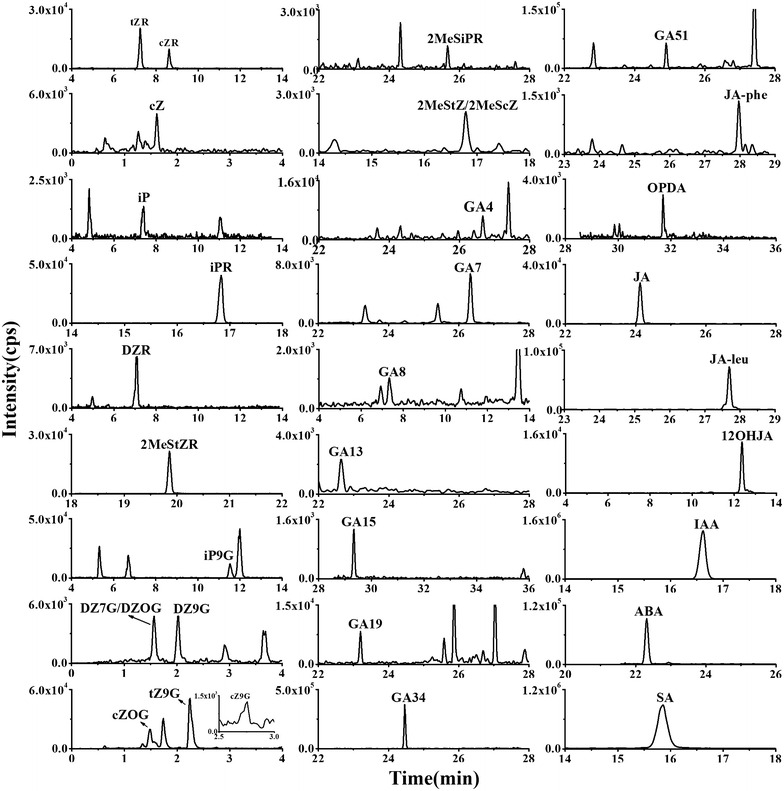
The chromatograms of detected phytohormones in rice ear at grain-filling stage

**Fig. 4:**
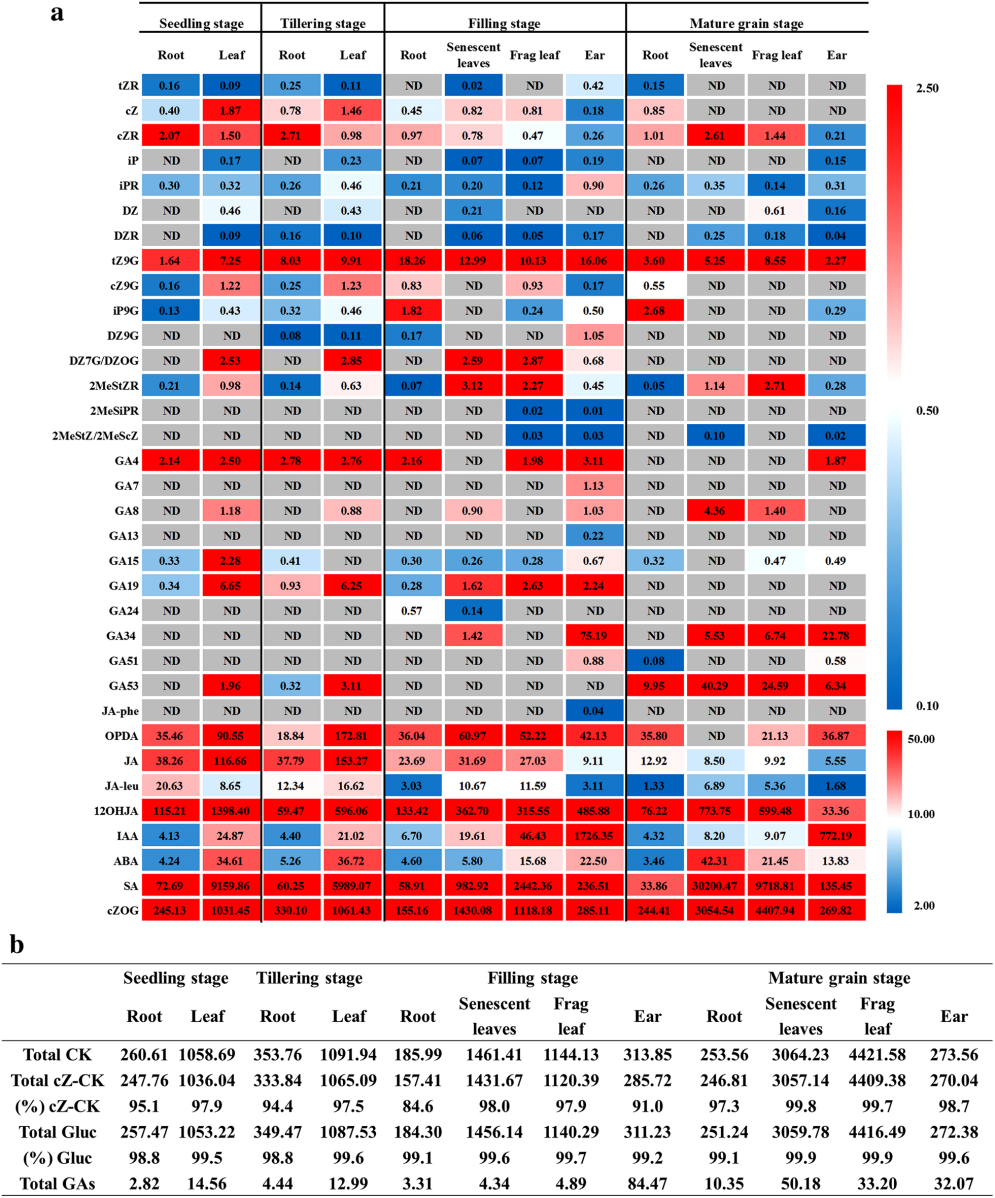
Spatiotemporal distribution of phytohormones in rice. **a** Heat map of spatiotemporal distribution of phytohormones. *Red* and *blue colors* indicate higher and lower concentrations, respectively. The *color scale* is shown at the *right*. Phytohormone species whose concentrations were under the quantification limit in all organs are not shown in the heat map. The value in each block is the concentration (average value, *n* = 3) as ng g^−1^ FW. *ND* not detected under the quantification limit. See Additional file 1: Table S6 for original data of measurement results. **b** Total amount of cytokinins (Total CK), cZ-type cytokinins (Total cZ-CK), cytokinin glucosides (Total gluc), and gibberellins (Total GAs) in the results of A are shown as ng g^−1^ FW. The proportions of cZ-type cytokinins [(%) cZ-CK] and cytokinin glucosides [(%) Gluc] are indicated as percentage values

To evaluate the spatiotemporal distribution of phytohormone species in rice. Root and leaves of rice (cv. ‘Zhenshan 97B’) plants were harvested at seedling stage and tillering stage. Root, senescent leaves, frag leaf and ear were harvested at grain-filling stage and mature grain stage. Then the endogenous hormone contents were analyzed. Among the 54 phytohormones investigated, 36 were quantified, including 18 CK species, 10 GA species, 5 JA species, IAA, ABA, and SA. The measurement results are shown in Additional file 1: Table S6. And the examples of chromatograms of rice tissue (rice ear at filling stage) are shown in Fig. 3. Accumulation of phytohormones displayed substantial variation in their abundance in different tissues of rice at different stages, as shown in the heat map (Fig. 4a). More species of hormones were detected in ear of rice at grain-filling stage than in other tissues, of which most showed higher concentrations. These indicated that phytohormones play important roles in seed development of rice [35].

**Fig. 5:**
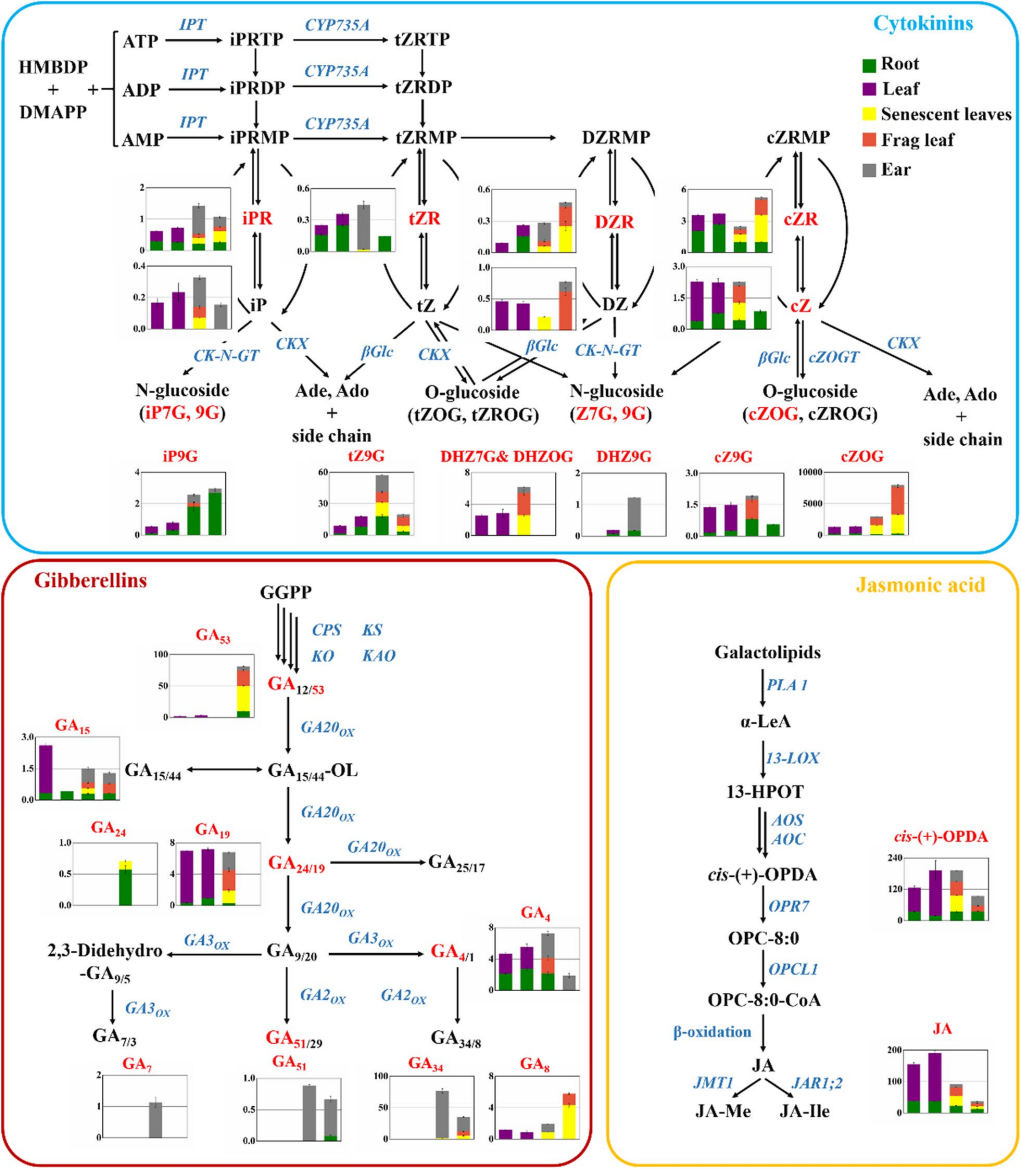
Endogenous levels of cytokinins, gibberellins and jasmonates in rice tissues at seedling stage, tillering stage, grain-filling stage and mature grain stage. The amounts of the hormones are shown as histograms with the SD (n = 3). The *y-axis* is concentration as ng g^−1^ FW. The *x-axis* represents the four growth stages. The details of each metabolic pathway are described by Hirano et al. [9]

In terms of cytokinins, cis-zeatin (cZ)-type cytokinins were dominant in all rice tissues investigated at all growth stages (Fig. 4b). The most abundant CK metabolite detected was cisZ-O-glucoside (cZOG) (Fig. 4a), being consistent with the previous reports [8, 36]. Glucosides were the major form of accumulated cytokinins in all tissues investigated, which are inactive and are thought to play a role in homeostasis of the hormones [1]. To better explain the dynamic change of endogenous levels of phytohormone species in rice, a metabolic pathway was shown in Fig. 5. In root, DZR was only detected at tillering stage. Contents of iP9G increased as the plants grown up. tZ9G, cZ9G and DZ9G increased from seedling stage to filling stage, but declined at mature grain stage. In frag leaf, cytokinin precursors such as DZR and cZR accumulated in frag leaf at mature grain stage. However, glucosides decreased in frag leaf when the rice grown mature, except for tZ9G and cZOG. For ear, almost all the cytokinins investigated decreased in the mature ear, except for DZ.

As for gibberellins, distinct tissue-specific accumulation patterns were observed. GA_7_, GA_51_ and GA_34_ were mainly accumulated in ear. Bioactive GA_4_ was detected in most of the tissues investigated except for senescent leaves at filling stage and tissues at mature grain stage. GA_7_ was only detected in ear of rice at filling stage. For root, GA precursor GA_53_ and downstream GA_51_ accumulated in root at mature grain stage. Bioactive GA_4_ showed significant reduction in root when the rice grown mature. For frag leaf, GA precursor GA_53_ and downstream GA_8_ accumulated at mature grain stage. For ear, GA precursor GA_53_ accumulated in mature ear, while the downstream GAs decreased, including the bioactive GA_4_ and GA_7_, and the deactivated GA_8_. GA_19_ accumulated in ear at grain-filing stage, but decreased to a very low level in mature ear, being consistent with the result Suzuki reported [37]. The decrease in GA_19_ content at mature grain stage may indicate vigorous consumption of GA_19_, which acts as a pool GA in the biosynthetic pathway to GA_7_, GA_51_, GA_34_ and GA_8_, which highly accumulated in ear at grain-filing stage.

The rice tissues also contained large amounts of ABA, IAA, OPDA, JA and SA. ABA showed higher concentrations in leaf than in root at seedling stage and tillering stage, and the content kept steady. From grain-filling stage to mature grain stage, ABA increased in senescent leaves, but did not change in the other tissues. IAA showed the lowest accumulation in root. However, an extremely high accumulation in ear was observed. The high accumulation of IAA in ear is consistent with the highly expressed genes related to auxin biosynthetic and metabolic processes, polar auxin transport, homeostasis and auxin-mediated signaling [35]. Concentration of JA declined in all tissues as the plants grown mature. Taken together, these results indicate that our analysis could show the spatiotemporal distribution pattern of the phytohormones in rice, and that the phytohormones are differentially distributed in rice tissues at different growth stages. However, for the further understanding of phytohormone function, some important clues obtained by transcriptome and other omics are needed.

## Conclusions

In this study, we have developed a rapid one-step method for the simultaneous analysis of six groups of phytohormones, including cytokinins, auxins, salicylic acid, jasmonates, abscisic acid and gibberellins in a single run, using UPLC–ESI–MS/MS. The proposed method was successfully applied to investigate spatiotemporal distribution of multiple phytohormones in rice. The spatiotemporal information obtained may be helpful for better understanding of phytohormones functions throughout life cycle of rice when integrated into transcriptome and other omics data.

## Methods

### Chemicals and reagents

Phytohormones standards: indole-3-acetic acid (IAA), indole-3-butyric acid (IBA), abscisic acid (ABA), salicylic acid (SA), jasmonic acid (JA), 2H-jasmonic acid (2H-JA) Jasmonic acid-leucine (JA-Ieu), Jasmonic acid-phenylalanine (JA-phe), 12-OH-jasmonic acid (12-OH-JA), 12-oxophytodienoic acid (OPDA), gibberellins (GA_1_, GA_3_, GA_4_, GA_5_, GA_6_, GA_7_, GA_8_, GA_9_, GA_12_, GA_13_, GA_15_, GA_19_, GA_20_, GA_23_, GA_24_, GA_29_, GA_34_, GA_44_, GA_51_, GA_53_); trans-zeatin (tZ), cis-zeatin (cZ), transzeatin-7-glucoside (tZ7G), trans-zeatin-9-glucoside (tZ9G), cis-zeatin-9-glucoside (cZ9G), cis-zeatin-O-glucoside (cZOG), dihydrozeatin (DZ), dihydrozeatin-7-glucoside (DZ7G), dihydrozeatin-9-glucoside (DZ9G), dihydrozeatin-O-glucoside (DZOG), isopentenyladenine (iP), N^6^-isopentenyladenine-7-glucoside (iP7G), N^6^-isopentenyladenine 9-glucoside (iP9G), trans-zeatin-riboside (tZR), cis-riboside (cZR), dihydrozeatin riboside (DZR), isopentenyladenine riboside (iPR), 2-chloro-trans- zeatin (2CltZ), 2-methylthio-trans-zeatin (2MeStZ), 2-methylthio-cis-zeatin (2MeScZ), 2-methylthio-trans-zeatin-riboside (2MeStZR), 2-methylthio-cis-zeatin-riboside (2MeScZR), 2-methylthio-N^6^-isopentenyladenine (2MeSiP), 2-methylthio-N^6^- isopentenyladenine riboside (2MeSiPR), 2-benzylthio-N^6^-isopentenyladenine (2BSiP) and stable isotope-labeled standards: [^2^H_2_]IAA, [^2^H_6_]ABA, [^2^H_4_]SA, [^2^H_2_]GA_1_, [^2^H_2_]GA_4_, [^2^H_2_]GA_5_, [^2^H_2_]GA_6_, [^2^H_2_]GA_7_, [^2^H_2_]GA_8_, [^2^H_2_]GA_9_, [^2^H_2_]GA_12_, [^2^H_2_]GA_15_, [^2^H_2_]GA_20_, [^2^H_2_]GA_24_, [^2^H_2_]GA_34_, [^2^H_2_]GA_44_, [^2^H_2_]GA_51_, [^2^H_2_]GA_53_, [^2^H_5_]tZ, [^15^N_4_]cZ, [^2^H_5_]tZ7G, [^2^H_5_]tZ9G, [^2^H_3_]DZ, [^2^H_5_]DZ9G, [^2^H_7_]DZOG, [^2^H_6_]iP, [^2^H_6_]iP9G, [^2^H_5_]tZR, [^2^H_3_]DZR, [^2^H_6_]iPR were all purchased from Olchemim Ltd. (Olomouc, Czech Republic). Acetonitrile (ACN, HPLC grade) was obtained from Tedia Co. (Fairfield, OH, USA). Ultra-pure water used throughout the study was purified with Milli-Q system (Milford, MA, USA). Formic acid (FA, 88%) was purchased from Sinopharm Chemical Reagent (Shanghai, China). Graphitized carbon black (GCB) was purchased from BOSHI Biotechnology Co., Ltd (shanghai, china, http://www.boshibio.com.cn).

### Plant materials

Rice (*Oryza sativa* ssp. indica cv. Zhenshan 97B) (kindly provided by Dr. Qian Qian from State Key Laboratory of Rice Biology, China National Rice Research Institute) plants were grown under natural field conditions during the rice-growing season (from June to October). Root and leaves were harvested at seedling stage and late-tillering stage. Root, senescent leaves, frag leaf and ear were harvested at grain-filling stage and mature grain stage. All the samples were harvested at 10:00–12:00 h, placed in liquid nitrogen immediately, and stored at −80 °C. Samples were taken from three different plants per line for three biological replicates.

### Sample pretreatment

As shown in Fig. 2, plant tissues (root, leaf and ear) (50 mg FW) were frozen with liquid nitrogen, grounded into powder with liquid nitrogen and then transferred into a 1.5-mL centrifuge tube. [^2^H_2_]IAA (1.0 ng), [^2^H_6_]ABA (1.0 ng), 2H-JA (1.0 ng) [^2^H_4_]SA (50 ng), [^2^H_2_]GA_1_ (0.5 ng), [^2^H_2_]GA_4_ (0.5 ng), [^2^H_2_]GA_5_ (0.5 ng), [^2^H_2_]GA_6_ (0.5 ng), [^2^H_2_]GA_7_ (0.5 ng), [^2^H_2_]GA_8_ (0.5 ng), [^2^H_2_]GA_9_ (0.5 ng), [^2^H_2_]GA_12_ (0.5 ng), [^2^H_2_]GA_15_ (0.5 ng), [^2^H_2_]GA_20_ (0.5 ng), [^2^H_2_]GA_24_ (0.5 ng), [^2^H_2_]GA_34_ (0.5 ng), [^2^H_2_]GA_44_ (0.5 ng), [^2^H_2_]GA_51_ (0.5 ng), [^2^H_2_]GA_53_ (0.5 ng), [^2^H_5_]tZ (0.1 ng), ^15^N_4_-cZ (0.1 ng), [^2^H_5_]tZ7G (0.1 ng), [^2^H_5_]tZ9G (0.1 ng), [^2^H_3_]DZ (0.1 ng), [^2^H_5_]DZ9G (0.1 ng), [^2^H_7_]DZOG (0.1 ng), [^2^H_6_]iP (0.1 ng), [^2^H_6_]iP9G (0.1 ng), [^2^H_5_]tZR (0.1 ng), [^2^H_3_]DZR (0.1 ng), [^2^H_6_]iPR (0.1 ng) mixture (in 5 μL ACN) was quickly added to the samples to serve as internal standards (I.S.) for the quantification. Then ACN (0.5 mL) was added and the mixture was vortexed for 30 s. After standing at −20 °C for 12 h, the supernatant was collected upon centrifugation at 10,000×*g* under 4 °C for 20 min. Subsequently, the supernatant was evaporated to dryness under a mild nitrogen stream at 35 °C and redissolved in 0.5 mL ACN containing 80% ACN (v/v). The sample solution was added into a 1.5-mLvial containing 10 mg graphitized carbon black. The mixture was vortexed vigorously for 3 min and the supernatant was transported to a 1.5-mL vial followed by evaporating to dryness. The residues were redissolved in 5% ACN (v/v) (50 μL) and 10 μL was injected for UPLC–MS/MS analysis.

### Instruments and analytical conditions

Analysis of phytohormones was performed on a UPLC–ESI (+/−)–MS/MS system consisting of a AB SCIEX 4500 triple quadrupole mass spectrometer (Foster City, CA, USA) with an electrospray ionization source (Turbo Ionspray), a Shimadzu LC-30AD.

HPLC system (Tokyo, Japan) with two 30AD pumps, a SIL-30AC auto sampler, a CTO-30A thermostat column compartment, and a DGU-20A5R degasser. Data acquisition and processing were performed using AB SCIEX Analyst 1.6 software (Foster City, CA, USA).

The HPLC separation was performed on a on a Shim-pack XR-ODS Ш column (75 mm × 2.0 mm i.d., 1.6 μm) purchased from Shimadzu (Tokyo, Japan) at 40 °C. A 52-min gradient of 0.1% FA (A) and ACN (B) was employed for the separation with a flow rate of 0.4 mL/min. A gradient programme of 4 min 5–5% B, 6 min 5–7% B, 10 min 7–20% B, 20 min 20–80% B, 2 min 80–5% and 5 min 5% B was used.

Multiple reaction monitoring (MRM) and the appropriate product ions were chosen to quantify phytohormones (Additional file 1: Table S2). The optimized conditions of MRM experiments were as follows: curtain gas, 40 psi; ion spray voltage, 5000 V for positive ion mode and −4500 V for negative ion mode; turbo heater temperature (TEM), 500 °C; nebulizing gas (Gas 1), 55 psi; heated gas (Gas 2), 40 psi. Data acquisition, peak integration, and the calculations were performed using Analyst 1.6.1 software (AB Sciex).

## Additional file

**Additional file 1:** **Figure S1.** Investigation of DSPE conditions. **Table S1.** Representative analytical methods for simultaneous determination of multiple phytohormones. **Table S2.** Summary of precursor-to-product ion transitions used for the quantification of phytohormones using UPLC-ESI-MS/MS. **Table S3.** The absolute recoveries of phytohormones extracted by GCB. **Table S4.** Linearities, LODs and LOQs of 54 phytohormones by GCB-based MSPE-UPLC-MS/MS method. **Table S5.** Precisions (intra- and inter-day) and recoveries of 54 phytohormones by GCB-based MSPE-UPLC-MS/MS method. **Table S6.** Contents of detected endogenous phytohormones in rice tissues.
