# Supplementary material for: A rapid approach to investigate spatiotemporal distribution of phytohormones in rice
Source: Plant Methods. 2016 Nov 17;12:47. doi: 10.1186/s13007-016-0147-1 (PMC5112728; doi:10.1186/s13007-016-0147-1)
Supplement: Supplementary file 1 — Additional file 1: Figure S1. Investigation of DSPE conditions. Table S1. Representative analytical methods for simultaneous determination of multiple phytohormones. Table S2. Summary of precursor-to-product ion transitions used for the quantification of phytohormones using UPLC-ESI-MS/MS. Table S3. The absolute recoveries of phytohormones extracted by GCB. Table S4. Linearities, LODs and LOQs of 54 phytohormones by GCB-based MSPE-UPLC-MS/MS method. Table S5. Precisions (intra- and inter-day) and recoveries of 54 phytohormones by GCB-based MSPE-UPLC-MS/MS method. Table S6. Contents of detected endogenous phytohormones in rice tissues. [file 13007_2016_147_MOESM1_ESM.docx]

**Supplementary Online Materials**

**A rapid approach to** **investigate spatiotemporal distribution of phytohormones in rice**

Wen-Jing Cai^╪^, Tian-Tan Ye^╪^, Qing Wang, Bao-Dong Cai, Yu-Qi Feng*

Key Laboratory of Analytical Chemistry for Biology and Medicine (Ministry of Education), Department of Chemistry, Wuhan University, Wuhan 430072, P.R. China

E-mail address for Wen-Jing Cai: [903415333@qq.com](mailto:903415333@qq.com); Tian-Tan Ye: 398644048@ qq.com; Qing Wang: [819966037@qq.com](mailto:819966037@qq.com) and Bao-Dong Cai: [852800776@qq.com](mailto:852800776@qq.com).

**Contents:**

**Figure S1.** Investigation of DSPE conditions.

**Table S1** Representative analytical methods for simultaneous determination of multiple phytohormones

**Table S2** Summary of precursor-to-product ion transitions used for the quantification of phytohormones using UPLC-ESI-MS/MS

**Table S3** The absolute recoveries of phytohormones extracted by GCB

**Table S4** Linearities, LODs and LOQs of 54 phytohormones by GCB-based MSPE-UPLC-MS/MS method.

**Table S5** Precisions (intra- and inter-day) and recoveries of 54 phytohormones by GCB-based MSPE-UPLC-MS/MS method.

**Table S6** Contents of detected endogenous phytohormones in rice tissues.

**
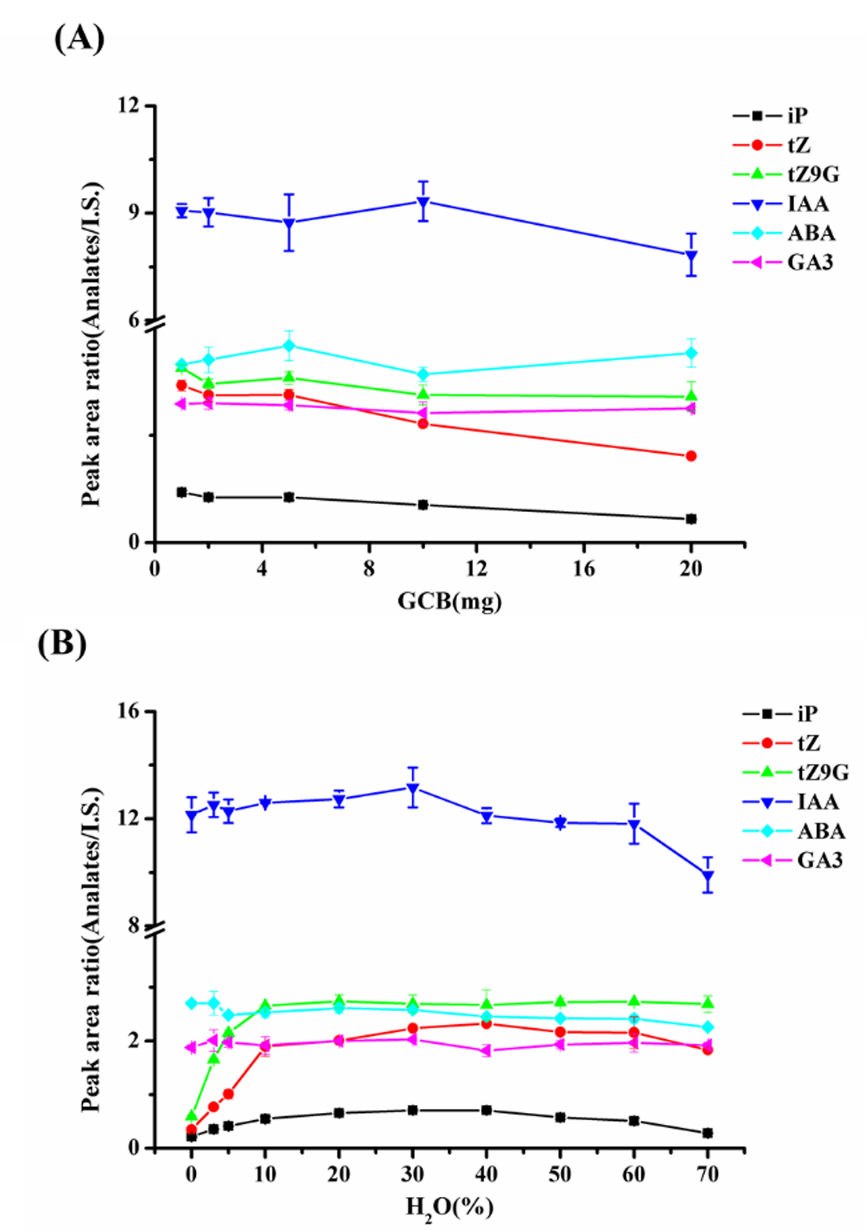
**

**Figure S1.** Investigation of GCB amount (A) and H2O content in sampling solution (B), Standards were spiked at 0.2 ng mL^−1^ for iP, tZ and tZ9G, 2 ng mL^−1^ for IAA, ABA, and GA_3_. Internal standards (I.S.) were added into the enriched samples just before UPLC-MS/MS analysis.

**Table S1** Representative analytical methods for simultaneous determination of multiple phytohormones.

| Sample matrix | Sample amount | Analytes | Sample preparation | | Final analysis | Method validation | Ref. |
| --- | --- | --- | --- | --- | --- | --- | --- |
|  |  |  | Extraction solvent | Purification method |  |  |  |
| Rice tissues | <100 mg | IAA, CTKs, ABA, GAs, and their metabolites (43 molecular species) | methanol/formic acid/water:15/1/4, v/v/v | (1) SPE using Oasis MCX 96-well plate;  (2) SPE using HLB 96-well plate | UPLC–ESI–qMS/MS | Recoveries were 7.0–111.7% | [1] |
| Rice seedlings | 200 mg | IAA, ABA, JA, SA, CKs, GAs, and their metabolites (43 molecular species) | 80% methanol | (1) SPE using PAX cartridges;  (2) SPE using PCX cartridges; | LC–MS/MS | LODs were 0.19- 7.57  fmol for IAA, GAs, ABA and their metabolites, 29.7 fmol for JA, 18.1 fmol for SA, and 0.03- 0.31 fmol for CKs and their metabolites. | [2] |
| Tissues of Arabidopsis thaliana, rice, grape, poplar and maize | 0.25- 1 g | IAA, ABA, GAs, and CTKs (24 molecular species) | 80% methanol | (1) SPE using MCX cartridges;  (2) SPE using MAX cartridges; | HPLC–ESI–MSn | LODs were 172.65 fmol for IAA, 13.78 and 65.84 fmol for cis-ABA and trans-ABA, around 100 fmol for GAs and under 1 fmol for most CTKs. | [3] |
| Tobacco dry seeds,  Arabidopsis seedlings | 10 mg for dry seeds, 100 mg  for fresh  seedlings | IAAs, CTKs, ABA, GAs (14 molecular species) | methanol/  formic acid/ water:  75/20/5, v/v/v | (1) SPE using Oasis HLB  cartridges;  (2) SPE using Oasis  MCX cartridges | nano-LC–ESI–IT-MS/MS | Recoveries for whole tissue sample were 8–95% with HLB and MCX cartridges. LOQs were 0.066–18 fmol | [4] |
| Grape Berry | 50 mg | ABAs, IAAs, and CKs (18 molecular species) | Methanol/formic acid/water: 15/1/4, v/v/v | (1) SPE using Oasis HLB  cartridges;  (2) SPE using  Oasis MCX cartridges | LC-MS/MS | 20% ion suppression for cytokinin-derived compounds | [5] |
| Nicotiana plants | 1.5 g | IAA, CTKs, ABA (6 molecular species) | methanol/  formic acid/water:  75/20/5, v/v/v | (1) SPE using Sep-Pak Plus C18 cartridges;  (2) SPE using Oasis MCX cartridges | LC–MS/MS and GC–MS | For LC–MS/MS method, recoveries were 87–98%; LODs were 0.5– 5 pg g^−1^. For GC-MS method, recoveries were 53–89%; LODs were 10–150 pg g^−1^ | [6] |
| Oryza sativa and Arabidopsis thaliana | 5 mg | IAA, ABA, JA, SA, iP, iPR, iP9G, tZ, tZR, tZ9G, DZ, DZR | ACN | Sequential SIPTE  (1) LLE using 0.1% formic acid (v/v)  (2) LLE using 50 mM NaHCO3 aqueous solution | UPLC–MS/MS | LODs were 0.56-438.60  pg mL^−1^. | [7] |
| pear flowers | 300 mg | GA4, ABA, IAA, **t**-ZR | 2-propanol/ H2O/ concentrated HCl: 2/10.002, v/v/v | 1. LLE using dichloromethane 2. SPE using Sep-Pak C18 cartridge | UPLC/ESI-MS/MS | Recoveries were 70.11-89.84%. The absolute ME values were 85.28–96.81%. LOQs were 0.26–3 ng mL^-1^ | [8] |
| Arabidopsis tissues | 50 mg | IAA, CTKs, ABA,  JAs, GAs, SA,  and their metabolites  (18 molecular species) | 2-Propanol/ H2O/ conc. HCl: 2/1/0.002, v/v/v | LLE using dichloromethane | HPLC–ESI–MS/MS | Recoveries were 85–98%. LOQs were 0.01-10 pg g^−1^ | [9] |
| Arabidopsis tissues | 50 mg | ZA, ABA, SA, JA, and BR | Methanol/acetic acid: 99/1, v/v | 0.45 μM nylon syringe filter | UFLC-MS/MS | Recoveries were 82.6-93.1 %. Signal suppression/ enhancement were 88.01-105.7 %. LODs were 0.04-0.49 ng mL^−1^ | [10] |
| Tomato plants | 100mg | GA_3_, ABA, IAA, JA, SA, Z, BA  and BL | Methanol/H2O/formic acid:  75/20/5, v/v/v | 30kDa Amicon® Ultra centrifugal filter unit | U-HPLC–Orbitrap-MS | LOQs were 0.18–1.41 pg μL^−1^ | [11] |
| Rosemary | 100 mg | SA, ABA, JA, IAA, GA_1_, GA_4_, GA_9_, GA_19_, GA_20_, GA_24_, ACC, 2iP, IPA, Z, ZR, DZ and DZR | Methanol/isopropanol/ glacial acetic: 20/80/1 (v/v/v) | filtration through 0.22 m PTFE filter | UPLC/ESI-MS/MS | LOQs were 0.09-12.10 ng/g DW | [12] |
| Lettuce seeds | 50–100 mg | IAA, CTKs, ABA, GAs, and their related metabolities (15 molecular species) | 2-Propanol/acetic acid: 99/1, v/v | SPE using Sep-Pak C18 cartridge | LC–ESI–MS/MS | Recoveries were greater than 80% except for d5-IAA (56%) and d3-DPA (65%). LODs were 0.682–1.53 fmol | [13] |
| Rice seedlings | 100 mg | 8 CKs, IAA, ABA, JA and10 GAs | ACN | MSPE using Fe3O4@TiO2 | UPLC-MS/MS | Recoveries were 53.2%-85.4%. LOQs were 0.02-0.38 fmol for CKs, 5.74, 6.58 and 0.55 fmol for IAA, ABA and JA, 0.21-3.92 fmol for GAs | [14] |
| Rice tissues | 50 mg | IAA, ABA, JA, SA, CKs, GAs, and their metabolites (54 molecular species) | ACN | DSPE using GCB | UPLC-MS/MS | LODs were 0.06- 9.17  fmol for IAA, JA, GAs, ABA and their metabolites, 31.1 fmol for SA, and 0.02- 9.97 fmol for CKs and their metabolites | This  work |

**Table S2** Summary of precursor-to-product ion transitions used for the quantification of phytohormones using UPLC-ESI-qMS/MS.

| Analytes | MRM^a^ function^b^ | RT^c^ (min) | Transition | IS^d^ | MRM function | RT (min) | Transition |
| --- | --- | --- | --- | --- | --- | --- | --- |
| tZ | 1 | 1.19 | 220.1>136.0 | d5-tZ | 1 | 1.16 | 225.1>137.0 |
| tZR | 2 | 7.18 | 352.2>136.0 | d5-tZR | 2 | 7.15 | 357.2>137.0 |
| cZ | 1 | 1.51 | 220.1>136.0 | ^15^N_4_-cZ | 1 | 1.48 | 224.0>140.0 |
| cZR | 2 | 8.65 | 352.2>220.0 | d5-tZR | 2 | 7.15 | 357.2>137.0 |
| iP | 2 | 7.31 | 204.1>136.0 | d6-iP | 2 | 7.33 | 210.1>137.0 |
| iPR | 3 | 16.80 | 336.1>204.1 | d6-iPR | 3 | 16.77 | 342.1>210.1 |
| DZ | 1 | 1.26 | 222.1>136.0 | d3-DZ | 1 | 1.23 | 225.2>136.0 |
| DZR | 2 | 7.05 | 354.1>222.0 | d3-DZR | 2 | 7.02 | 357.2>225.1 |
| tZ7G | 1 | 1.15 | 382.1>220.1 | d5-tZ7G | 1 | 1.07 | 387.1>225.1 |
| tZ9G | 1 | 2.20 | 382.1>220.1 | d5-tZ9G | 1 | 2.18 | 387.1>225.1 |
| cZ9G | 1 | 2.73 | 382.1>220.1 | d5-tZ9G | 1 | 2.69 | 387.1>225.1 |
| cZOG | 1 | 1.44 | 382.1>220.1 | ^15^N_4_-cZ | 1 | 1.48 | 224.0>140.0 |
| DZ7G | 1 | 1.47 | 384.2>222.0 | d7-DZOG | 1 | 1.45 | 391.2>229.1 |
| DZ9G | 1 | 2.15 | 384.2>222.0 | d5-DZ9G | 1 | 2.13 | 387.2>225.1 |
| DZOG | 1 | 1.47 | 384.2>222.0 | d7-DZOG | 1 | 1.45 | 391.2>229.1 |
| iP7G | 2 | 4.64 | 366.0>204.1 | d6-iP | 2 | 4.62 | 210.1>137.0 |
| iP9G | 2 | 11.50 | 366.1>204.1 | d6-iP9G | 2 | 11.47 | 372.2>210.1 |
| 2BSiP | 6 | 30.20 | 326.2>90.9 | d6-iPR | 3 | 16.77 | 342.1>210.1 |
| 2CltZ | 3 | 16.60 | 254.2>170 | d6-iPR | 3 | 16.77 | 342.1>210.1 |
| 2MeStZ | 3 | 16.80 | 265.9>182.0 | d6-iPR | 3 | 16.77 | 342.1>210.1 |
| 2MeScZ | 3 | 16.80 | 265.9>182.0 | d6-iPR | 3 | 16.77 | 342.1>210.1 |
| 2MeStZR | 4 | 19.90 | 398>265.9 | d6-iPR | 3 | 16.77 | 342.1>210.1 |
| 2MeScZR | 4 | 20.00 | 398>265.9 | d6-iPR | 3 | 16.77 | 342.1>210.1 |
| 2MeSiP | 5 | 24.70 | 250.0>182.0 | d6-iPR | 3 | 16.77 | 342.1>210.1 |
| 2MeSiPR | 5 | 25.60 | 381.9>250.0 | d6-iPR | 3 | 16.77 | 342.1>210.1 |
| GA_1_ | 3 | 16.90 | 347.1>272.7 | d2-GA1 | 3 | 16.87 | 349.1>274.7 |
| GA_3_ | 3 | 16.60 | 345.2>142.6 | d2-GA1 | 3 | 16.87 | 349.1>274.7 |
| GA_4_ | 5 | 26.70 | 331.1>256.7 | d2-GA4 | 5 | 26.67 | 333>258.7 |
| GA_5_ | 5 | 22.80 | 329.1>144.6 | d2-GA5 | 5 | 22.77 | 331.1>144.6 |
| GA_6_ | 4 | 19.60 | 345.1>118.6 | d2-GA6 | 4 | 19.57 | 347.1>118.6 |
| GA_7_ | 5 | 26.40 | 329.0>222.7 | d2-GA7 | 5 | 26.36 | 331.1>224.7 |
| GA_8_ | 2 | 7.42 | 363.1>274.7 | d2-GA8 | 2 | 7.39 | 365.1>276.7 |
| GA_9_ | 6 | 29.40 | 315.0>270.6 | d2-GA9 | 6 | 29.37 | 317.1>272.8 |
| GA_12_ | 6 | 30.90 | 331.0>312.9 | d2-GA12 | 6 | 30.87 | 333.1>315.1 |
| GA_13_ | 5 | 22.70 | 377.1>302.8 | d2-GA20 | 5 | 23.17 | 333.1>288.8 |
| GA_15_ | 6 | 29.30 | 329.1>130.6 | d2-GA15 | 6 | 29.27 | 331.1>132.7 |
| GA_19_ | 5 | 23.20 | 361.1>272.8 | d2-GA19 | 5 | 23.17 | 363.1>272.7 |
| GA_20_ | 5 | 23.20 | 331.3>286.8 | d2-GA20 | 5 | 23.17 | 333.1>288.8 |
| GA_23_ | 3 | 14.20 | 377.1>276.7 | d2-GA1 | 3 | 16.87 | 349.1>274.7 |
| GA24 | 5 | 27.30 | 345.1>256.7 | d2-GA24 | 5 | 27.27 | 347.2>258.8 |
| GA29 | 2 | 9.30 | 347.1>240.7 | d2-GA8 | 2 | 7.39 | 365.1>276.7 |
| GA34 | 5 | 24.50 | 347.1>258.7 | d2-GA34 | 5 | 24.46 | 349.1>260.8 |
| GA44 | 5 | 23.70 | 345.1>254.7 | d2-GA44 | 5 | 23.67 | 347.1>184.6 |
| GA51 | 5 | 24.90 | 331.0>286.8 | d2-GA51 | 5 | 24.88 | 333.1>288.8 |
| GA53 | 5 | 25.50 | 347.1>302.9 | d2-GA53 | 5 | 25.47 | 349.0>304.9 |
| JA | 5 | 24.10 | 209.0>58.8 | H2-JA | 5 | 25.98 | 211.1>58.9 |
| JA-leu | 5 | 27.80 | 322.1>129.6 | H2-JA | 5 | 25.98 | 211.1>58.9 |
| JA-phe | 5 | 28.10 | 356.1>163.5 | H2-JA | 5 | 25.98 | 211.1>58.9 |
| 12OHJA | 2 | 13.20 | 225.1>58.8 | d2-GA8 | 2 | 7.39 | 211.1>58.9 |
| OPDA | 6 | 31.70 | 291.1>164.6 | d2-GA12 | 6 | 30.87 | 333.1>315.1 |
| IAA | 3 | 16.68 | 176.1>130.1 | d2-IAA | 3 | 16.64 | 178.1>132.0 |
| IBA | 5 | 24.20 | 204.2>186.1 | d6-ABA | 5 | 22.27 | 269.1>224.7 |
| ABA | 5 | 22.30 | 263.0>218.6 | d6-ABA | 5 | 22.27 | 269.1>224.7 |
| SA | 3 | 15.90 | 137.0>92.7 | d4-SA | 3 | 15.88 | 141.0>96.7 |

^a^ MRM, multiple reaction monitoring

^b^ In order to enhance the sensitivity of UPLC-ESI-MS/MS, six separate functions were implemented in the MRM mode so that only ions eluted during the specified retention windows were monitored.

^C^ RT, retention time; ^d^ IS, internal standard.

**Table S3** The absolute recoveries of plant hormones extracted by GCB (n = 3).

| Recovery% | tZ | | tZR | | | cZ | | cZR | | iP | | | iPR | DZ | DZR | tZ7G |
| --- | --- | --- | --- | --- | --- | --- | --- | --- | --- | --- | --- | --- | --- | --- | --- | --- |
| In ACN/H2O | 49.16±0.67 | | 67.86±0.77 | | | 40.29±1.19 | | 62.12±2.91 | | 33.52±1.81 | | | 61.24±1.80 | 48.06±2.25 | 70.54±1.00 | 86.33±4.04 |
| In matrix | 69.01±7.10 | | 81.47±1.91 | | | 62.53±0.90 | | 70.18±3.17 | | 65.25±2.80 | | | 66.68±0.96 | 66.72±3.02 | 71.63±0.46 | 97.51±4.19 |
|  |  |  | |  |  | |  |  |  | |  |  |  |  |  |  |
| Recovery% | tZ9G | | cZ9G | | | cZOG | | DZ7G | | DZ9G | | | iP7G | iP9G | 2BSiP | 2CltZ |
| In ACN/H2O | 77.22±2.40 | | 73.1±3.42 | | | 61.03±0.87 | | 88.27±4.13 | | 74.63±2.32 | | | 88.13±2.60 | 80.36±0.71 | 27.41±1.28 | 34.16±0.49 |
| In matrix | 88.15±4.94 | | 72.44±1.04 | | | 97.78±4.42 | | 98.75±0.63 | | 70.18±3.01 | | | 86.33±4.84 | 82.89±7.99 | 78.53±3.37 | 59.05±3.31 |
|  |  | |  | | |  | |  | |  | | |  |  |  |  |
| Recovery% | 2MeStZ | | 2MeStZR | | | 2MeScZR | | 2MeSiP | | 2MeSiPR | | | GA1 | GA3 | GA4 | GA5 |
| In ACN/H2O | 21.83±1.02 | | 44.69±1.39 | | | 38.77±1.14 | | 11.87±0.10 | | 20.95±0.22 | | | 92.39±0.97 | 91.38±1.11 | 84.19±0.26 | 68.98±1.72 |
| In matrix | 77.47±1.11 | | 98.83±4.47 | | | 76.39±0.49 | | 40.32±1.73 | | 94.6±5.30 | | | 88.74±8.56 | 83.56±2.17 | 91.72±6.94 | 74.9±1.85 |
|  |  | |  | | |  | |  | |  | | |  |  |  |  |
| Recovery% | GA6 | | GA7 | | | GA8 | | GA9 | | GA12 | | | GA13 | GA15 | GA19 | GA20 |
| In ACN/H2O | 77.4±5.03 | | 81.31±1.41 | | | 79.88±4.11 | | 79.36±0.44 | | 71.88±1.24 | | | 78.1±4.01 | 71.34±2.82 | 98.14±3.88 | 79.44±1.42 |
| In matrix | 87.98±3.86 | | 79.13±2.35 | | | 96.15±1.67 | | 92.07±7.03 | | 83.3±3.65 | | | 97.76±2.9 | 76.61±1.33 | 106.79±3.01 | 83.98±2.37 |
|  |  | |  | | |  | |  | |  | | |  |  |  |  |
| Recovery% | GA23 | | GA24 | | | GA29 | | GA34 | | GA44 | | | GA51 | GA53 | OPDA | 12OHJA |
| In ACN/H2O | 87.79±4.51 | | 85.1±1.46 | | | 76.16±3.58 | | 94.8±0.84 | | 59.74±2.81 | | | 81.08±0.72 | 102.84±4.81 | 32.34±1.74 | 84.02±2.47 |
| In matrix | 96±5.05 | | 80.61±4.24 | | | 88.21±4.02 | | 82.91±3.27 | | 77.71±3.54 | | | 105.03±4.14 | 105.04±5.52 | 68.15±3.58 | 90.62±4.13 |
|  |  | |  | | |  | |  | |  | | |  |  |  |  |
| Recovery% | IAA | | JA-leu | | | JA-phe | | IBA | | ABA | | | JA | SA |  |  |
| In ACN/H2O | 87.06±2.83 | | 103.86±10.02 | | | 95.07±4.08 | | 48.93±2.23 | | 77.97±1.87 | | | 84.17±4.43 | 89.65±4.08 |  |  |
| In matrix | 109.03±2.60 | | 92.76±5.20 | | | 101.04±9.74 | | 75.78±1.97 | | 98.8±2.36 | | | 84.47±3.33 | 99.01±5.21 |  |  |

**Table S4** Linearities, LODs and LOQs of 54 plant hormones by GCB-based MSPE-UPLC-MS/MS method.

| Analytes | Linear dynamic range (ng/ml) | Regression line | | | LODs (fmol) | LOQs (fmol) |
| --- | --- | --- | --- | --- | --- | --- |
|  |  | slope | Intercept | r value |  |  |
| tZ | 0.01-100 | 0.9431 | 0.0782 | 0.9999 | 0.14 | 0.41 |
| tZR | 0.01-100 | 0.7940 | -0.0353 | 0.9995 | 0.09 | 0.26 |
| cZ | 0.01-100 | 0.4324 | 0.1112 | 0.9999 | 0.13 | 0.38 |
| cZR | 0.01-50 | 1.4224 | 0.1440 | 0.9994 | 0.07 | 0.22 |
| iP | 0.01-100 | 0.1647 | -0.0193 | 0.9990 | 0.17 | 0.50 |
| iPR | 0.005-50 | 0.2142 | 0.0231 | 0.9993 | 0.02 | 0.07 |
| DZ | 0.01-100 | 0.4010 | -0.0117 | 0.9991 | 0.13 | 0.38 |
| DZR | 0.01-100 | 0.3443 | 0.1271 | 0.9994 | 0.08 | 0.24 |
| tZ7G | 0.005-100 | 0.0616 | 0.0253 | 0.9992 | 0.04 | 0.12 |
| tZ9G | 0.05-100 | 0.3183 | 0.1022 | 0.9992 | 0.13 | 0.39 |
| cZ9G | 0.05-100 | 0.4714 | 0.1099 | 0.9994 | 0.12 | 0.36 |
| cZOG | 2-1000 | 0.0033 | -0.0098 | 0.9999 | 9.97 | 29.92 |
| DZ9G | 0.01-100 | 0.5519 | 0.2067 | 0.9992 | 0.07 | 0.22 |
| DZOG | 0.01-100 | 1.3269 | 0.8595 | 0.9987 | 0.08 | 0.24 |
| iP7G | 0.01-100 | 0.1100 | -0.0163 | 1.0000 | 0.06 | 0.18 |
| iP9G | 0.01-50 | 0.5946 | 0.0457 | 0.9999 | 0.10 | 0.29 |
| 2BSiP | 0.2-500 | 0.0003 | -0.0002 | 0.9996 | 1.44 | 4.33 |
| 2CltZ | 0.02-100 | 0.0463 | 0.0113 | 0.9997 | 0.15 | 0.45 |
| 2MeStZ | 0.005-100 | 0.6371 | 0.0050 | 0.9987 | 0.02 | 0.06 |
| 2MeStZR | 0.005-100 | 0.1574 | 0.0649 | 0.9995 | 0.02 | 0.06 |
| 2MeScZR | 0.005-100 | 0.1038 | 0.0247 | 0.9997 | 0.04 | 0.12 |
| 2MeSiP | 0.01-100 | 0.3000 | 0.0195 | 0.9996 | 0.16 | 0.47 |
| 2MeSiPR | 0.01-50 | 0.3064 | 0.0128 | 0.9998 | 0.04 | 0.11 |
| GA_1_ | 0.01-100 | 0.1150 | 0.2151 | 0.9964 | 0.06 | 0.18 |
| GA_3_ | 0.05-100 | 0.3644 | 0.2280 | 0.9996 | 0.19 | 0.58 |
| GA_4_ | 0.1-100 | 0.0774 | 0.0547 | 0.9992 | 0.44 | 1.33 |
| GA_5_ | 0.05-50 | 0.0983 | 0.0587 | 0.9980 | 0.26 | 0.77 |
| GA_6_ | 0.1-100 | 0.0980 | 0.0691 | 0.9985 | 0.22 | 0.65 |
| GA_7_ | 0.05-50 | 0.0802 | 0.0039 | 0.9999 | 0.56 | 1.68 |
| GA_8_ | 0.5-100 | 0.0726 | 0.0557 | 0.9989 | 4.47 | 13.42 |
| GA_9_ | 0.1-50 | 0.0899 | -0.0060 | 0.9994 | 1.09 | 3.28 |
| GA_12_ | 0.5-50 | 0.0247 | -0.0183 | 0.9996 | 2.23 | 6.68 |
| GA_13_ | 0.05-50 | 0.7745 | 0.0380 | 0.9999 | 0.37 | 1.11 |
| GA_15_ | 0.05-50 | 0.1099 | 0.0342 | 0.9999 | 0.16 | 0.48 |
| GA_19_ | 0.05-50 | 0.7331 | 0.0190 | 1.0000 | 0.39 | 1.16 |
| GA_20_ | 0.1-50 | 0.0735 | 0.0194 | 0.9998 | 0.89 | 2.66 |
| GA_23_ | 0.2-100 | 0.0261 | 0.0161 | 0.9983 | 1.51 | 4.52 |
| GA_24_ | 0.05-50 | 0.1994 | 0.0329 | 0.9997 | 0.22 | 0.67 |
| GA_29_ | 1-100 | 0.0093 | 0.0151 | 0.9980 | 9.17 | 27.50 |
| GA_34_ | 0.05-50 | 0.0282 | -0.0019 | 0.9996 | 0.46 | 1.37 |
| GA_44_ | 0.2-100 | 0.2073 | -0.0212 | 0.9995 | 2.07 | 6.22 |
| GA_51_ | 0.3-50 | 0.0807 | 0.0487 | 0.9991 | 1.73 | 5.19 |
| GA_53_ | 0.05-50 | 0.0803 | -0.0011 | 0.9981 | 0.37 | 1.12 |
| JA | 0.05-1000 | 0.0498 | 0.0015 | 1.0000 | 0.37 | 1.12 |
| JA-leu | 0.01-1000 | 0.3041 | 0.1491 | 0.9993 | 0.08 | 0.24 |
| JA-phe | 0.01-1000 | 0.2038 | 0.0755 | 0.9994 | 0.09 | 0.26 |
| 12OHJA | 0.2-1000 | 0.0413 | -0.0079 | 0.9996 | 1.73 | 5.20 |
| OPDA | 0.5-1000 | 0.0164 | -0.0011 | 0.9989 | 4.02 | 12.07 |
| IBA | 0.2-1000 | 0.0444 | 0.0364 | 0.9994 | 2.69 | 8.06 |
| ABA | 0.5-1000 | 0.1042 | 0.0364 | 0.9990 | 2.68 | 8.03 |
| IAA | 0.03-1000 | 0.0406 | 0.0185 | 0.9997 | 4.29 | 12.88 |
| SA | 10-10000 | 0.0228 | 0.0516 | 0.9997 | 31.09 | 93.26 |

**Table S5** Precisions (intra- and inter-day) and recoveries of 54 phytohormones by GCB-based MSPE-UPLC-MS/MS method.

| Analytes | Intra-day precision  (RSD%, n=5) | | | Inter-day precision  (RSD%, n=3) | | | Recovery  (%, n = 5) | | |
| --- | --- | --- | --- | --- | --- | --- | --- | --- | --- |
|  | Low | Medium | High | Low | Medium | High | Low | Medium | High |
| Added (ng/g) | 1 | 5 | 25 | 1 | 5 | 25 | 1 | 5 | 25 |
| tZ | 5.0 | 3.8 | 3.4 | 4.8 | 4.4 | 3.2 | 100.0 | 94.4 | 88.6 |
| tZR | 11.3 | 1.2 | 2.9 | 4.3 | 1.7 | 2.3 | 100.0 | 93.2 | 85.9 |
| cZ | 8.7 | 5.0 | 4.4 | 6.6 | 4.4 | 5.0 | 97.6 | 103.2 | 99.1 |
| cZR | 5.8 | 3.4 | 3.9 | 7.1 | 10.2 | 3.9 | 97.3 | 96.1 | 96.8 |
| tZ7G | 6.9 | 4.4 | 10.0 | 5.8 | 0.8 | 3.4 | 90.3 | 90.7 | 95.1 |
| tZ9G | 7.4 | 4.1 | 0.7 | 11.8 | 5.2 | 3.5 | 86.0 | 89.4 | 99.0 |
| cZ9G | 8.4 | 0.8 | 5.4 | 11.1 | 7.3 | 5.8 | 90.8 | 89.5 | 97.6 |
| DHZ | 7.4 | 2.5 | 1.8 | 7.5 | 5.5 | 6.4 | 104.0 | 90.1 | 103.0 |
| DHZ7G | 10.5 | 4.9 | 4.6 | 9.8 | 3.5 | 4.5 | 113.7 | 117.5 | 109.2 |
| DHZ9G | 6.0 | 5.9 | 7.5 | 5.6 | 2.6 | 3.9 | 91.6 | 100.5 | 98.8 |
| DHZOG | 8.7 | 4.9 | 1.4 | 10.8 | 2.7 | 5.7 | 81.1 | 87.3 | 82.5 |
| DHZR | 9.6 | 11.5 | 11.6 | 1.8 | 2.9 | 2.7 | 94.5 | 97.8 | 99.3 |
| iP | 6.6 | 3.7 | 5.6 | 6.6 | 3.6 | 6.8 | 105.0 | 114.0 | 101.2 |
| iPR | 6.8 | 9.9 | 4.1 | 7.0 | 7.1 | 3.8 | 93.0 | 105.6 | 97.1 |
| iP7G | 9.4 | 4.7 | 3.7 | 10.8 | 5.4 | 6.7 | 91.6 | 100.5 | 98.8 |
| iP9G | 4.4 | 8.2 | 1.0 | 6.0 | 6.3 | 3.5 | 99.0 | 108.0 | 109.9 |
| 2CltZ | 6.1 | 3.5 | 7.9 | 1.9 | 9.1 | 4.0 | 86.2 | 95.0 | 90.3 |
| 2MeStZ/2MeScZ | 9.2 | 7.5 | 9.3 | 9.4 | 7.1 | 1.6 | 94.5 | 99.8 | 96.3 |
| 2MeStZR | 3.6 | 1.8 | 3.2 | 3.0 | 2.8 | 3.2 | 91.9 | 101.5 | 98.8 |
| 2MeScZR | 8.5 | 4.4 | 6.7 | 7.8 | 8.9 | 8.5 | 84.2 | 82.7 | 88.2 |
| 2MeSiP | 9.8 | 1.5 | 8.2 | 3.8 | 8.3 | 2.5 | 94.9 | 97.8 | 99.3 |
| 2MeSiPR | 4.1 | 3.2 | 5.4 | 3.7 | 5.2 | 2.7 | 100.4 | 92.1 | 93.9 |
| 2BSiP | 7.9 | 9.1 | 6.6 | 6.5 | 6.3 | 1.3 | 80.7 | 87.9 | 88.5 |
| Added (ng/g) | 20 | 50 | 200 | 20 | 50 | 200 | 20 | 50 | 200 |
| IAA | 7.9 | 1.7 | 3.5 | 6.1 | 4.8 | 3.9 | 120.0 | 114.8 | 106.0 |
| IBA | 6.8 | 0.9 | 1.7 | 7.1 | 3.4 | 5.9 | 91.6 | 100.5 | 98.8 |
| ABA | 10.1 | 3.9 | 4.6 | 3.8 | 3.5 | 4.5 | 117.0 | 120.0 | 107.0 |
| JA | 5.6 | 3.2 | 1.0 | 7.6 | 4.5 | 2.6 | 107.5 | 110.0 | 106.8 |
| JA-leu | 7.5 | 4.7 | 3.9 | 6.8 | 5.7 | 1.9 | 113.3 | 95.0 | 94.1 |
| JA-phe | 7.8 | 1.9 | 0.7 | 6.1 | 3.4 | 5.0 | 89.0 | 85.6 | 98.8 |
| 12OHJA | 9.1 | 3.7 | 6.6 | 7.8 | 3.5 | 1.5 | 81.1 | 87.3 | 82.5 |
| OPDA | 8.6 | 7.2 | 1.0 | 7.9 | 4.5 | 6.6 | 86.2 | 95.0 | 90.3 |
| Added (ng/g) | 300 | 500 | 1000 | 300 | 500 | 1000 | 300 | 500 | 1000 |
| SA | 11.8 | 6.7 | 8.9 | 10.9 | 7.3 | 6.5 | 102.8 | 111.7 | 108.9 |
| cZOG | 9.7 | 7.4 | 6.8 | 8.7 | 7.1 | 3.2 | 104.6 | 107.3 | 107.5 |
| Added (ng/g) | 2 | 5 | 20 | 2 | 5 | 20 | 2 | 5 | 20 |
| GA1 | 7.4 | 6.4 | 2.3 | 10.5 | 10.4 | 7.8 | 102.0 | 98.4 | 90.0 |
| GA3 | 10.8 | 3.7 | 4.2 | 11.2 | 9.4 | 9.1 | 104.0 | 99.2 | 109.7 |
| GA4 | 5.9 | 2.2 | 4.0 | 8.0 | 11.0 | 6.6 | 108.0 | 88.8 | 96.4 |
| GA5 | 5.9 | 5.3 | 2.0 | 11.3 | 10.5 | 9.6 | 103.0 | 106.0 | 117.1 |
| GA6 | 7.5 | 6.8 | 6.4 | 11.5 | 5.8 | 7.3 | 89.0 | 84.8 | 120.0 |
| GA7 | 4.0 | 2.2 | 2.4 | 5.6 | 4.9 | 5.0 | 96.0 | 94.8 | 107.7 |
| GA8 | 6.5 | 2.0 | 3.8 | 11.2 | 7.4 | 5.5 | 100.0 | 80.3 | 110.0 |
| GA9 | 5.5 | 7.5 | 7.0 | 8.7 | 8.9 | 6.7 | 111.0 | 104.0 | 120.4 |
| GA12 | 9.3 | 11.3 | 9.5 | 11.4 | 7.9 | 5.4 | 105.0 | 92.4 | 93.2 |
| GA13 | 8.8 | 5.0 | 6.2 | 7.4 | 4.0 | 1.3 | 94.5 | 97.8 | 99.3 |
| GA15 | 10.7 | 4.8 | 4.1 | 10.8 | 6.0 | 1.1 | 94.0 | 90.4 | 105.8 |
| GA19 | 11.0 | 5.6 | 2.1 | 11.1 | 3.4 | 7.6 | 89.0 | 85.6 | 98.8 |
| GA20 | 5.6 | 3.0 | 2.6 | 10.4 | 5.6 | 3.0 | 101.0 | 95.2 | 82.0 |
| GA23 | 10.7 | 5.2 | 6.8 | 9.5 | 1.9 | 3.7 | 84.2 | 82.7 | 88.2 |
| GA24 | 8.2 | 3.0 | 1.7 | 5.8 | 6.5 | 8.4 | 91.0 | 96.4 | 102.5 |
| GA29 | 5.0 | 6.5 | 1.0 | 9.1 | 3.0 | 4.1 | 113.3 | 95.0 | 94.1 |
| GA34 | 7.7 | 9.1 | 1.2 | 3.1 | 5.4 | 3.3 | 109.3 | 110.9 | 104.3 |
| GA44 | 9.4 | 8.0 | 5.5 | 11.8 | 4.3 | 10.7 | 83.0 | 111.6 | 120.0 |
| GA51 | 4.5 | 7.4 | 6.5 | 5.9 | 1.2 | 3.4 | 94.0 | 95.2 | 96.4 |
| GA53 | 4.5 | 3.8 | 3.6 | 6.2 | 8.4 | 5.5 | 110.0 | 82.5 | 85.0 |

**Table S6** Contents of detected endogenous phytohormones in rice tissues. (unit, ng g^−1^ FW).

|  | **Seedling stage** | | **Tillering stage** | | **Filling stage** | | | | **Mature grain stage** | | | |
| --- | --- | --- | --- | --- | --- | --- | --- | --- | --- | --- | --- | --- |
| Sample Name | Root | Leaf | Root | Leaf | Root | Senescent leaves | Frag leaf | Ear | Root | Senescent leaves | Frag leaf | Ear |
| tZR | 0.16  ±0.01 | 0.09  ±0.01 | 0.25  ±0.01 | 0.11  ±0.02 | ND | 0.02  ±0.001 | ND | 0.42  ±0.04 | 0.15  ±0.002 | ND | ND | ND |
| cZ | 0.40  ±0.001 | 1.87  ±0.12 | 0.78  ±0.05 | 1.46  ±0.19 | 0.45  ±0.04 | 0.82  ±0.06 | 0.81  ±0.03 | 0.18  ±0.01 | 0.85  ±0.07 | ND | ND | ND |
| cZR | 2.07  ±0.04 | 1.5  ±0.07 | 2.71  ±0.09 | 0.98  ±0.04 | 0.97  ±0.1 | 0.78  ±0.02 | 0.47  ±0.02 | 0.26  ±0.02 | 1.01  ±0.02 | 2.61  ±0.06 | 1.44  ±0.03 | 0.21  ±0.02 |
| iP | ND | 0.17  ±0.03 | ND | 0.23  ±0.06 | ND | 0.07  ±0.003 | 0.07  ±0.01 | 0.19  ±0.01 | ND | ND | ND | 0.15  ±0.01 |
| iPR | 0.30  ±0.004 | 0.32  ±0.01 | 0.26  ±0.02 | 0.46  ±0.03 | 0.21  ±0.01 | 0.2  ±0.01 | 0.12  ±0.01 | 0.9  ±0.06 | 0.26  ±0.03 | 0.35  ±0.004 | 0.14  ±0.01 | 0.31  ±0.03 |
| DZ | ND | 0.46  ±0.03 | ND | 0.43  ±0.03 | ND | 0.21  ±0.01 | ND | ND | ND | ND | 0.61  ±0.06 | 0.16  ±0.01 |
| DZR | ND | 0.09  ±0.01 | 0.16  ±0.01 | 0.1  ±0.01 | ND | 0.06  ±0.01 | 0.05  ±0.01 | 0.17  ±0.01 | ND | 0.25  ±0.05 | 0.18  ±0.01 | 0.04  ±0.003 |
| tZ9G | 1.64  ±0.07 | 7.25  ±0.58 | 8.03  ±0.13 | 9.91  ±0.23 | 18.26  ±1.59 | 12.99  ±0.48 | 10.13  ±0.74 | 16.06  ±0.11 | 3.6  ±0.2 | 5.25  ±0.03 | 8.55  ±0.43 | 2.27  ±0.11 |
| cZ9G | 0.16  ±0.03 | 1.22  ±0.04 | 0.25  ±0.01 | 1.23  ±0.14 | 0.83  ±0.06 | ND | 0.93  ±0.14 | 0.17  ±0.01 | 0.55  ±0.02 | ND | ND | ND |
| iP9G | 0.13  ±0.01 | 0.43  ±0.03 | 0.32  ±0.02 | 0.46  ±0.06 | 1.82  ±0.3 | ND | 0.24  ±0.01 | 0.5  ±0.05 | 2.68  ±0.15 | ND | ND | 0.29  ±0.03 |
| DZ9G | ND | ND | 0.08  ±0.005 | 0.11  ±0.01 | 0.17  ±0.01 | ND | ND | 1.05  ±0.01 | ND | ND | ND | ND |
| DZ7G/DZOG | ND | 2.53  ±0.12 | ND | 2.85  ±0.49 | ND | 2.59  ±0.12 | 2.87  ±0.3 | 0.68  ±0.03 | ND | ND | ND | ND |
| 2MeStZR | 0.21  ±0.01 | 0.98  ±0.05 | 0.14  ±0.01 | 0.63  ±0.04 | 0.07  ±0.005 | 3.12  ±0.2 | 2.27  ±0.1 | 0.45  ±0.03 | 0.05  ±0.004 | 1.14  ±0.04 | 2.71  ±0.11 | 0.28  ±0.02 |
| 2MeSiPR | ND | ND | ND | ND | ND | ND | 0.02  ±0.001 | 0.01  ±0.001 | ND | ND | ND | ND |
| 2MeStZ/2MeScZ | ND | ND | ND | ND | ND | ND | 0.03  ±0.002 | 0.03  ±0.001 | ND | 0.1  ±0.01 | ND | 0.02  ±0.003 |
| GA4 | 2.14  ±0.12 | 2.5  ±0.17 | 2.78  ±0.29 | 2.76  ±0.38 | 2.16  ±0.19 | ND | 1.98  ±0.26 | 3.11  ±0.29 | ND | ND | ND | 1.87  ±0.29 |
| GA7 | ND | ND | ND | ND | ND | ND | ND | 1.13  ±0.16 | ND | ND | ND | ND |
| GA8 | ND | 1.18  ±0.13 | ND | 0.88  ±0.13 | ND | 0.9  ±0.01 | ND | 1.03  ±0.02 | ND | 4.36  ±0.28 | 1.40  ±0.10 | ND |
| GA13 | ND | ND | ND | ND | ND | ND | ND | 0.22  ±0.01 | ND | ND | ND | ND |
| GA15 | 0.33  ±0.003 | 2.28  ±0.1 | 0.41  ±0.01 | ND | 0.3  ±0.02 | 0.26  ±0.01 | 0.28  ±0.05 | 0.67  ±0.06 | 0.32  ±0.004 | ND | 0.47  ±0.03 | 0.49  ±0.05 |
| GA19 | 0.34  ±0.02 | 6.65  ±0.07 | 0.93  ±0.02 | 6.25  ±0.2 | 0.28  ±0 | 1.62  ±0.15 | 2.63  ±0.24 | 2.24  ±0.08 | ND | ND | ND | ND |
| GA24 | ND | ND | ND | ND | 0.57  ±0.06 | 0.14  ±0.01 | ND | ND | ND | ND | ND | ND |
| GA34 | ND | ND | ND | ND | ND | 1.42  ±0.17 | ND | 75.19  ±3.41 | ND | 5.53  ±0.64 | 6.74  ±0.6 | 22.78  ±0.77 |
| GA51 | ND | ND | ND | ND | ND | ND | ND | 0.88  ±0.02 | 0.08  ±0.01 | ND | ND | 0.58  ±0.04 |
| GA53 | ND | 1.96  ±0.33 | 0.32  ±0.03 | 3.11  ±0.17 | ND | ND | ND | ND | 9.95  ±0.11 | 40.29  ±0.57 | 24.59  ±1.04 | 6.34  ±0.53 |
| JA-phe | ND | ND | ND | ND | ND | ND | ND | 0.04  ±0.003 | ND | ND | ND | ND |
| OPDA | 35.46  ±1.94 | 90.55  ±6.77 | 18.84  ±1.53 | 172.81  ±37.41 | 36.04  ±2.41 | 60.97  ±0.2 | 52.22  ±1.53 | 42.13  ±0.85 | 35.8  ±0.34 | ND | 21.13  ±1.38 | 36.87  ±1.69 |
| JA | 38.26  ±2.72 | 116.66  ±5.24 | 37.79  ±2.44 | 153.27  ±7.04 | 23.69  ±0.63 | 31.69  ±1.21 | 27.03  ±0.71 | 9.11  ±0.15 | 12.92  ±0.9 | 8.5  ±0.49 | 9.92  ±0.67 | 5.55  ±0.6 |
| JA-leu | 20.63  ±1.9 | 8.65  ±0.84 | 12.34  ±1.01 | 16.62  ±0.78 | 3.03  ±0.13 | 10.67  ±0.62 | 11.59  ±0.92 | 3.11  ±0.13 | 1.33  ±0.02 | 6.89  ±0.14 | 5.36  ±0.16 | 1.68  ±0.13 |
| 12OHJA | 115.21  ±13.02 | 1398.4  ±11.72 | 59.47  ±0.04 | 596.06  ±29.68 | 133.42  ±11.04 | 362.7  ±23.85 | 315.55  ±4.15 | 485.88  ±29.6 | 76.22  ±5.74 | 773.75  ±11.31 | 599.48  ±62.44 | 33.36  ±2.17 |
| IAA | 4.13  ±0.44 | 24.87  ±1.46 | 4.40  ±0.31 | 21.02  ±0.63 | 6.70  ±0.58 | 19.61  ±1.01 | 46.43  ±3.38 | 1726.35  ±25.52 | 4.32  ±0.47 | 8.2  ±1.82 | 9.07  ±0.47 | 772.19  ±29.42 |
| ABA | 4.24  ±0.06 | 34.61  ±1.37 | 5.26  ±0.57 | 36.72  ±0.27 | 4.6  ±0.42 | 5.8  ±0.14 | 15.68  ±1.17 | 22.5  ±0.72 | 3.46  ±2.45 | 42.31  ±3.82 | 21.45  ±1.06 | 13.83  ±0.4 |
| SA | 72.69  ±8.02 | 9159.86  ±31.29 | 60.25  ±10.98 | 5989.07  ±284.08 | 58.91  ±5.75 | 982.92  ±28.98 | 2442.36  ±36.67 | 236.51  ±24.46 | 33.86  ±3.25 | 30200.47  ±188.53 | 9718.81  ±350.71 | 135.45  ±4.15 |
| cZOG | 245.13  ±17.82 | 1031.45  ±83.17 | 330.1  ±28.66 | 1061.43  ±36.68 | 155.16  ±6.92 | 1430.08  ±23.52 | 1118.18  ±15.07 | 285.11  ±2.22 | 244.41  ±15.5 | 3054.54  ±42.53 | 4407.94  ±97.31 | 269.82  ±20.81 |

ND: not detected.

**REFERENCES**

1. Kojima M, Kamada-Nobusada T, Komatsu H, Takei K, Kuroha T, Mizutani M, Ashikari M, Ueguchi-Tanaka M, Matsuoka M, Suzuki K, Sakakibara H. Highly sensitive and high-throughput analysis of plant hormones using MS-probe modification and liquid chromatography-tandem mass spectrometry: an application for hormone profiling in Oryza sativa. Plant Cell Physiol, 2009; 50: 1201-14.

2. Cao Z Y, Sun L H, Mou R X, Zhang L P, Lin X Y, Zhu Z W, Chen M X. Profiling of phytohormones and their major metabolites in rice using binary solid-phase extraction and liquid chromatography-triple quadrupole mass spectrometry. J Chromatogr A, 2016.

3. Liu S, Chen W, Qu L, Gai Y, Jiang X. Simultaneous determination of 24 or more acidic and alkaline phytohormones in femtomole quantities of plant tissues by high-performance liquid chromatography–electrospray ionization–ion trap mass spectrometry. Analytical and Bioanalytical Chemistry, 2013; 405: 1257-1266.

4. Izumi Y, Okazawa A, Bamba T, Kobayashi A, Fukusaki E. Development of a method for comprehensive and quantitative analysis of plant hormones by highly sensitive nanoflow liquid chromatography–electrospray ionization-ion trap mass spectrometry. Analytica Chimica Acta, 2009; 648: 215-225.

5. Gouthu S, Morre J, Maier C S, Deluc L G. An Analytical Method to Quantify Three Plant Hormone Families in Grape Berry Using Liquid Chromatography and Multiple Reaction Monitoring Mass Spectrometry, in Phytochemicals, Plant Growth, and the Environment, R.D. Gang, Editor. 2013, Springer New York: New York, NY. p. 19-36.

6. Giannarelli S, Muscatello B, Bogani P, Spiriti M M, Buiatti M, Fuoco R. Comparative determination of some phytohormones in wild-type and genetically modified plants by gas chromatography-mass spectrometry and high-performance liquid chromatography-tandem mass spectrometry. Anal Biochem, 2010; 398: 60-8.

7. Cai B D, Ye E C, Yuan B F, Feng Y Q. Sequential solvent induced phase transition extraction for profiling of endogenous phytohormones in plants by liquid chromatography-mass spectrometry. J Chromatogr B Analyt Technol Biomed Life Sci, 2015; 1004: 23-29.

8. Niu Q, Zong Y, Qian M, Yang F, Teng Y. Simultaneous quantitative determination of major plant hormones in pear flowers and fruit by UPLC/ESI-MS/MS. Analytical Methods, 2014; 6: 1766-1773.

9. Pan X Q, Welti R, Wang X M. Quantitative analysis of major plant hormones in crude plant extracts by high-performance liquid chromatography-mass spectrometry. Nature Protocols, 2010; 5: 986-992.

10. Kasote D M, Ghosh R, Chung J Y, Kim J, Bae I, Bae H. Multiple Reaction Monitoring Mode Based Liquid Chromatography-Mass Spectrometry Method for Simultaneous Quantification of Brassinolide and Other Plant Hormones Involved in Abiotic Stresses. Int J Anal Chem, 2016; 2016: 7214087.

11. Van Meulebroek L, Bussche J V, Steppe K, Vanhaecke L. Ultra-high performance liquid chromatography coupled to high resolution Orbitrap mass spectrometry for metabolomic profiling of the endogenous phytohormonal status of the tomato plant. Journal of Chromatography A, 2012; 1260: 67-80.

12. Müller M, Munné-Bosc S. Rapid and sensitive hormonal profiling of complex plant samples by liquid chromatography coupled to electrospray ionization tandem mass spectrometry. Plant Methods, 2011; 7.

13. Chiwocha S D S, Abrams S R, Ambrose S J, Cutler A J, Loewen M, Ross A R S, Kermode A R. A method for profiling classes of plant hormones and their metabolites using liquid chromatography-electrospray ionization tandem mass spectrometry: an analysis of hormone regulation of thermodormancy of lettuce (Lactuca sativa L.) seeds. Plant Journal, 2003; 35: 405-417.

14. Cai B D, Yin J, Hao Y H, Li Y N, Yuan B F, Feng Y Q. Profiling of phytohormones in rice under elevated cadmium concentration levels by magnetic solid-phase extraction coupled with liquid chromatography tandem mass spectrometry. J Chromatogr A, 2015.
